# Prognostic value of neutrophil-to-lymphocyte ratio (NLR) in penile cancer: A systematic review and meta-analysis

**DOI:** 10.1016/j.amsu.2022.104335

**Published:** 2022-08-05

**Authors:** Haviv Muris Saputra, Furqan Hidayatullah, Yudhistira Pradnyan Kloping, Johan Renaldo, Eric Chung, Lukman Hakim

**Affiliations:** a Department of Urology, Faculty of Medicine, Universitas Airlangga, Surabaya, East Java, Indonesia; b Dr. Soetomo General-Academic Hospital, Surabaya, East Java, Indonesia; c Department of Urology, Princess Alexandra Hospital, University of Queensland, Brisbane, Qld, Australia; d Universitas Airlangga Teaching Hospital, Surabaya, East Java, Indonesia

**Keywords:** *Penile cancer*, *Neutrophil to lymphocyte ratio*, *Overall survival*, *Cancer-specific survival*, *Cancer*

## Abstract

**Background:**

Penile cancer is rare among male malignancies. Various biomarkers have been used to predict the prognosis of cancer, one of which is the neutrophil to lymphocyte ratio (NLR). Therefore, we conducted this systematic review and meta-analysis to evaluate the prognostic value of NLR in penile cancer.

**Methods:**

This review was conducted following the PRISMA guideline. Several databases, including Scopus, Science-direct, and PubMed, were systematically searched. The primary outcomes were lymph node metastasis (LNM), cancer-specific survival (CSS), and overall survival (OS). All statistical analyses were processed using Review Manager (RevMan) version 5.4.

**Results:**

A total of six retrospective studies were included in the analysis. The cut-off values of NLR in the included studies ranged from 2.6 to 3.59. Meta-analysis showed that penile cancer patients with high NLR had worse LNM and CSS based on the univariate analysis (OR 3.56, 95% CI 2.38, 5.32, p < 0.01; HR 4.19, 95% CI 2.19, 8.01, p = 0.0; respectively). Furthermore, the meta-analysis revealed that NLR is an independent predictor of LNM and CSS (OR 6.67, 95% CI 2.44, 18.22, p < 0.01; HR 2.15, 95% CI 1.23, 3.73, p < 0.01; respectively). However, NLR failed to show as independent predictor for OS (HR 1.69,95% CI 0.95,3.00, p = 0.07).

**Conclusion:**

NLR is an independent predictor of LNM and CSS. However, NLR is not proven to be an independent predictor of OS in this study.

## Highlights

• NLR is an independent predictor of LNM in penile cancer.
• NLR is an independent predictor of CSS in penile cancer.
• This study failed to demonstrate the association of NLR as predictor for OS.

## 1 Introduction

Penile cancer is rare among male malignancies and causes a substantial psychological effect on the patient. In the United States and Europe, penile cancer only accounts for 0.4–0.6% of malignant diagnoses [1,2]. On the contrary, the Brazilian state of Maranhão has the world's highest incidence of penile cancer (ASR of 6.1 cases per 100,000 people) [3]. Patients with inguinal lymph node involvement have a poor prognosis [4,5]. Predictors of inguinal lymph node metastasis consisted of the pathological stage of the primary tumor, grade, and lymphovascular invasion. Penile cancer is likely incurable once systemic metastasis has occurred. Local lymphatic dissemination to regional lymph nodes in the inguinal area occurs stepwise, with the superficial inguinal lymph nodes usually the first primary points of cancer metastasis [6].

Recent literature highlights that the association between systemic inflammation and tumor development is confirmed in various solid organ cancers [7]. Since complete blood count can signify underlying systemic inflammation, the Neutrophil to Lymphocyte Ratio (NLR) invariably serves as an indicator of tissue inflammation in cancer [8]. The NLR measures the systemic inflammatory and immunological responses. Several studies have evaluated the predictive use of pre-treatment NLR as an independent predictor of overall survival (OS) in patients with penile squamous cell carcinoma and inguinal lymph node. NLR also corresponds to the nodal stage of the tumor. Published data showed a positive association between systemic inflammation and survival/prognosis [[9], [10], [11], [12]]. This systematic review and meta-analysis evaluated the prognostic value of NLR in penile cancer.

## 2 Methods

### 2.1 Study design

This systematic review and meta-analysis study was conducted by following the guideline of PRISMA (Preferred Reporting Items for Systematic Reviews and Meta-Analyses) [13]. Self-evaluation of this study was assessed and in accordance with AMSTAR 2 criteria [14]. The study protocol was registered in PROSPERO (CRD42022271381) and research registry (reviewregistry1415).

### 2.2 Systematic Search strategy

A comprehensive online literature search was performed to select the potential studies on PubMed, Scopus, and ScienceDirect library database inception of December 2021. The following keyword was used (“NLR” or “Neutrophils-to-Lymphocyte Ratio” or “neutrophil to lymphocyte ratio”) and (“penile cancer” or “carcinoma of the penis” or “malignancy of penis”). A complete search strategy for included studies is provided in Table 1.

**Table 1:** Keywords used as literature search strategy.

Search-engine	Keywords	Article(n)
PubMED	(“neutrophil-to-lymphocyte ratio"[All Fields] OR “neutrophil-to-lymphocyte ratio"[All Fields] OR “nlr"[All Fields]) AND (((“penil"[All Fields] OR “penis"[MeSH Terms] OR “penis"[All Fields] OR “penile"[All Fields]) AND (“carcinoma"[MeSH Terms] OR “carcinoma"[All Fields] OR “carcinomas"[All Fields] OR “carcinoma s"[All Fields])) OR (“penile neoplasms"[MeSH Terms] OR (“penile"[All Fields] AND “neoplasms"[All Fields]) OR “penile neoplasms"[All Fields] OR (“penile"[All Fields] AND “cancer"[All Fields]) OR “penile cancer"[All Fields]) OR (“penile neoplasms"[MeSH Terms] OR (“penile"[All Fields] AND “neoplasms"[All Fields]) OR “penile neoplasms"[All Fields] OR (“penis"[All Fields] AND “neoplasms"[All Fields]) OR “penis neoplasms"[All Fields]))	12
Scopus	TITLE-ABS-KEY ((“neutrophil two lymphocyte ratio” OR “neutrophil-two-lymphocyte ratio” OR nlr) AND (penile AND carcinoma OR penile AND cancer OR penis AND neoplasms))	18
Science-direct	(“neutrophil to lymphocyte ratio” OR “neutrophil-to-lymphocyte ratio” OR nlr) AND (penile carcinoma OR penile cancer OR Penis neoplasms)	92
		122

### 2.3 Eligibility criteria

The study enrolled in this systematic review and meta-analysis should meet these inclusion criteria: (1) studies which compared penile cancer patients with high NLR to patients with low NLR before surgery to determine predictor of oncological and metastatic outcomes; (2) Required data can be extracted; (3) Publication articles were available on PubMed, ScienceDirect, and Scopus database. The studies were excluded following these exclusion criteria: (1) case report, case series, and article review, (2) In vitro research, (3) animal studies, and (4) unpublished article.

### 2.4 Data extraction and quality assessment

Three investigators (HMS, FH and YPK) independently extracted the following items: study characteristics (authors, years of publication, location of studies, sample size); baseline characteristics of the sample (type of histopathology, age of the samples, modality of treatment, and follow up duration); NLR cut-off values; cut off values methods; outcome (lymph node metastasis (LNM), cancer-specific survival (CSS), and overall survival (OS). The Newcastle-Ottawa Scale (NOS), which includes selection, comparability, and exposure, was used to assess the risk of bias in each enrolled literature [15]. The score classification was described in 0–3 as a low-quality study, while 4–6 as a medium quality study, and 7–9 as a high-quality study. When there were discrepancies between the two investigators, the decision was made after being discussed with the third investigator.

The outcomes of this study were LNM, CSS, and OS. LNM was calculated from the result of the clinicopathology data. Pooled Odd Ratio (OR) was extracted directly if reported in the studies. In survival analysis, the HRs and 95% confidence intervals (CIs) were extracted and used to calculate pooled hazard ratio (HR). I^2^ statistics were used to assess the heterogeneity among the included studies. If significant heterogeneity existed (I^2^ >50% and/or p < 0.10), the pooled HRs and 95% CIs were calculated by a random-effect model; otherwise, the fixed-effect model was performed (I^2^ < 50% and/or p > 0.10). All the analysis was performed with RevMan 5.4 for windows.

## 3 Results

### 3.1 Systematic Search results

**Fig. 1:**
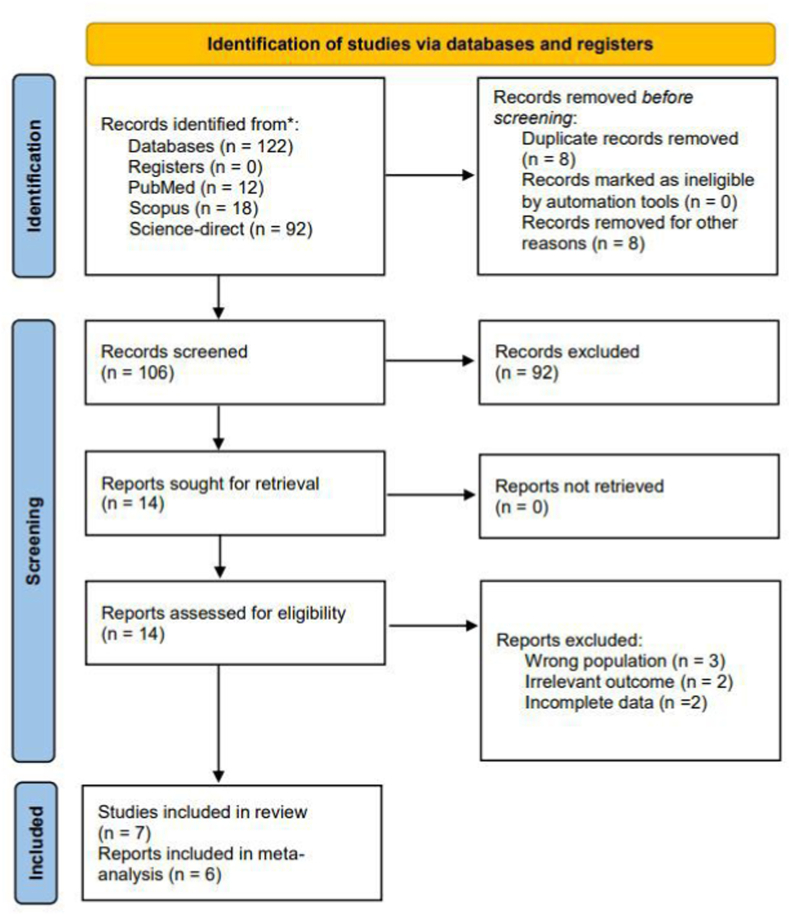
Systematic Search and Screening based on the 2020 PRISMA flow diagram.

PRISMA Flow diagram [13] (Fig. 1) demonstrates the article searching and selection process. Our initial search from multiple databases yielded a total of 122 records. Sixteen articles were removed due to duplicates and other reasons (non-original article and animal study), leaving 106 articles to be screened through the Mendeley reference manager. After the primary screening process, fourteen studies were further evaluated in full text. The final analysis included seven articles [7,[9], [10], [11], [12],^16^,^17^] for the systematic review, six of which [7,[9], [10], [11], [12],^16^] were eligible to be included in the meta-analysis.

### 3.2 Study characteristics

All the included studies were single-institutional retrospective cohorts, except for a multi-institutional retrospective cohort by Li et al. [16]. Studies were mostly conducted in the Asian population, except for Azizi et al. [10], which was conducted in the American population. Participants from the included studies had an average age ranging from 50.6 years to 68.2 years. The median study follow-up ranged from 18 months to 35.5 months, as summarized in Table 2. All the participants in the included studies had undergone inguinal lymph node dissection (ILND), with pathological confirmation of penile squamous cell carcinoma (SCC). Table 3 summarizes the outcome assessment in the included studies, while Table 4 summarizes the results of the oncological outcome for penile SCC with high NLR compared to low NLR. Studies used different cut-off values to determine high and low NLR, ranging from 2.6 to 3.59. There were various methods for determining optimal NLR cut-off values, including the area under the curve (AUC), receiver operating characteristic (ROC), and Contal and O'Quigley methods. The median OS was not described in many of the included studies, and only Azizi et al. [10] and Chen Hu et al. [12] reported the median patients' OS of 89 months and 34 months, respectively.

**Table 2:** Baseline characteristics of the included study.

No	Author & years of publication	Years of Data Collection	Patient's TNM Staging	Country	Study Design	Total patients (n)	Group Allocation	Allocated patients (n)	Cancer Pathology	Age in years (mean ± SD)	Follow-up duration
1	Kasuga et al., 2016	1999–2015	T1, T2, T3, T4 N0, N + M0, M+	Japan	Single Institutional Retrospective Cohort	41	High NLR	20	Penile SCC	68.5 ± 11.4	34.7 (2.3–271.7) months^b^
							Low NLR	21			
2	Tan et al., 2017	2007–2015	Ta, T1a, T1b, T2, T3	Singapore	Single Institutional Retrospective Cohort	39	High NLR	NR	Penile SCC	65. (59–72.5)^b^	34 (16.5–66) months^b^
			N0, N1, N2, N3				Low NLR	NR			
3	Azizi et al., 2018	1994–2014	Tx, Tis/Ta/T1a, T1b, T2, T3, T4	USA	Single Institutional Retrospective Cohort	84	High NLR	38	Penile SCC	63.6(54–68.7)^a^ 68.2(53.4–73.6)^a^	35.5 (19.4–89.6) months^b^
			N0, N+				Low NLR	30			
4	Li et al., 2019	2002–2015	≤T1, ≥T2	China	Multi- Institutional Retrospective cohort	228	High NLR	105	Penile SCC	52(24–85)^a^	25 (1–140) months^a^
			N0, N+				Low NLR	123			
5	Jiao Hu et al., 2020	2010–2018	Tis, Ta/T1a, ≥T1b	China	Single Institutional Retrospective Cohort	134	High NLR	32	Penile SCC	54.95 ± 10.6	32.1(2–94) month^a^
			No, N+				Low NLR	47			
6	Chen Hu et al., 2020	2002–2017	T0, T1, T2, T3, T4	China	Single Institutional Retrospective Cohort	225	High NLR	68	Penile SCC	50.6 ± 13.4	30 (16–63.5) months^b^
			N0, N1, N2, N3 M0, M1				Low NLR	157			
7	Jindal et al., 2021	2012–2020	T1, T2, T3, T4	India	Single Institutional Retrospective Cohort	69	High NLR	40	Penile SCC	NR	18 (2–74) months^a^
			N0, N1, N2, N3				Low NLR	29			

a Data expressed as median and range.

b Data expressed as median and interquartile (IQR) range, SCC = Squamous cell carcinoma.

**Table 3:** Outcome assessment and treatment received in the included studies.

No	Author & years of publication	Surgical Intervention	NLR (mean ± SD)	NLR Cut-off	Cut-off point calculation	Median OS	Median CSS	Primary Outcome	Secondary Outcome
1	Kasuga et al., 2016	Radical Penectomy	5.03 ± 4.99	2.82	AUC	NR	NR	OS, CSS	LNM
2	Tan et al., 2017	Bilateral modified inguinal lymph node dissection or dynamic sentinel node biopsy	2.99 (0.76–5.22)∗∗	2.8	AUC	NR	NR	CSS, PFS,	LNM
3	Azizi et al., 2018	Bilateral/unilateral inguinal lymph node dissection	4.51 ± 3.95	3	Contal and O'Quigley method	89 (31.2–123.6) months∗∗	NR	OS, CSS, RFS	LNM
4	Li et al., 2019	Bilateral inguinal lymph node dissection	2.4 (0.1–44.2)∗	2.6	ROC	NR	NR	CSS	LNM
5	Jiao Hu et al., 2020	Bilateral inguinal lymph node dissection after primary tumor procedure	NR	3.59	ROC	NR	NR	CSS	LNM
6	Chen Hu et al., 2020	Inguinal lymph node dissection	NR	2.94	AUC	34 (18–84) months∗∗	33 (18–85.25) months∗∗	OS, PFS	LNM
7	Jindal et al., 2021	Penectomy with bilateral inguinal node dissection	NR	3	Following previous study by Azizi et al.	NR	18 (2–74) months∗	CSS	LNM

NLR = Neutrophil-lymphocyte ratio, ROC = Receiver operating characteristic curve, AUC = Area under the curve, OS = Overall Survival, CSS = Cancer specific-survival, PFS = Progression-free survival, LNM = Lymph-Node Metastasis.

∗ Data expressed as median and range.

∗∗ Data expressed as median and interquartile (IQR) range.

**Table 4:** Oncological outcomes results summary for penile SCC with high NLR compared to low NLR.

Outcome	Analysis methods	Reference	Result	*p* value
Overall Survival	Univariate Analysis	Kasuga et al., 2016	NR	0.076
		Chen Hu et al., 2020	HR: 2.97 (95% CI 1.74 to 5.06)	<0.001
	Multivariate Analysis	Chen Hu et al., 2020	HR: 1.28 (95% CI 0.61 to 2.72)	0.516
		Azizi et al., 2018	HR: 2.48 (95% CI 1.02 to 6.03)	0.046
Cancer-Specific Survival	Univariate Analysis	Kasuga et al., 2016	NR	0.023
		Azizi et al., 2018	HR: 6.16 (95% CI 2.1 to 18.07)	0.014
		Jiao Hu et al., 2020	HR: 3.36 (95% CI 1.49 to 7.58)	<0.01
		Jindal et al., 2021	NR	0.05
	Multivariate Analysis	Tan et al., 2017	NR	<0.01
		Azizi et al., 2018	HR: 2.58 (95% CI 0.79 to 8.43)	0.116
		Li et al., 2019	HR: 2.25 (95% CI 1.12 to 4.51)	0.023
		Jiao Hu et al., 2020	HR: 1.27 (95% CI 0.28 to 5.70)	0.76
		Jindal et al., 2021	NR	0.94
Lymph-Node Metastasis	Univariate Analysis	Azizi et al., 2018	OR: 3.75 (95% CI 1.30 to 10.81)	0.014
		Chen Hu et al., 2020	OR: 2.21 (95% CI 1.23 to 3.99)	0.008
		Jiao Hu et al., 2020	OR: 6.92 (95% CI 2.46 to 19.43)	<0.01
		Jindal et al., 2021	OR: 6.13 (95% CI 2.13 to 17.65)	0.001
		Kasuga et al., 2016	OR: 5.12 (95% CI 0.91 to 28.64)	0.049
	Multivariate Analysis	Azizi et al., 2018	OR: 3.66 (95% CI 0.82 to 16.34)	0.091
		Jiao Hu et al., 2020	OR: 10.93 (95% CI 2.81 to 42.51)	<0.01
		Jindal et al., 2021	NR	0.09

OR = Odds ratio, HR = Hazard Ratio, NR = Not reported.

Additionally, Chen Hu et al. [12] found that patients with high NLR had a shorter OS than patients with low NLR, with a median of 30 months and 158 months, respectively. In addition, the median CSS was also not described in the results of most of the studies. However, Chen Hu et al. [12] and Jindal et al. [7] reported that the penile cancer patients in their cohort had a median CSS of 33 months and 18 months, respectively. The value of NLR was obtained from a complete blood count examination taken from peripheral blood samples prior to the surgical procedure which the course varied from 1 month to 3 days before inguinal lymph node dissection surgery.

### 3.3 Risk of bias

All the included studies in the meta-analysis were retrospective cohorts, and the quality of these studies was assessed using the NOS [15], which was designed explicitly for observational studies. This scale evaluates each article's selection, comparability, and outcome domain. Since available data from medical records and control populations were selected from the same population as the exposed population, all studies were scored with a good score in the NOS selection domain. Most studies had controlled for various factors that could affect the outcomes such as age, based on multivariate analysis and fulfilled a good comparability score on NOS. Similarly, most of the included articles in this review had reported adequate follow-up duration and description, so the NOS outcome domain was also good. In general, the overall NOS evaluation revealed that all the included studies had a good quality, as presented in Table 5.

**Table 5:** Risk of bias assessment using the Newcastle-Ottawa Scale.

Authors	Selection	Comparability	Outcome	Total Score
Tindal et al., 2021	***	**	**	7
Hu Jiao et al., 2020	***	**	***	8
Zaishang et al., 2020	****	**	***	9
Hu Chen et al., 2020	***	**	***	8
Azizi et al., 2018	****	**	***	9
Kasuga et al., 2016	***	**	***	8

### 3.4 Meta-analysis results on the lymph node invasion

**Fig. 2:**
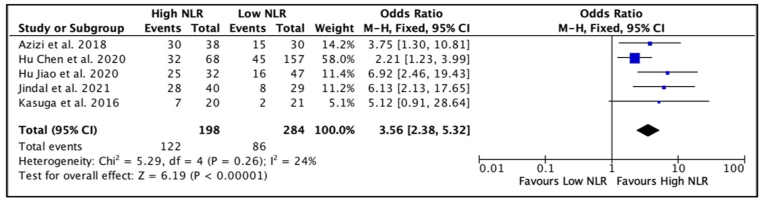
Forest plot comparison of unadjusted OR of node invasion in penile cancer patients with high NLR versus low NLR.

**Fig. 3:**
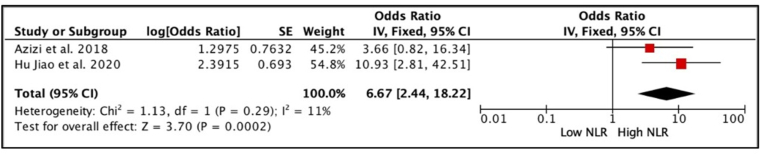
Forest plot comparison of adjusted OR of lymph node invasion in penile cancer patients with high NLR versus low NLR.

The meta-analysis included five studies [7,[9], [10], [11], [12]], totalling 482 patients with penile cancer who underwent inguinal node dissection. Based on the forest plot analysis, patients with high NLR values had a higher incidence of lymph node invasion compared to patients with low NLR (OR 3.56, 95% CI 2.38, 5.32, p < 0.01) (Fig. 2). Furthermore, a meta-analysis performed on the multivariate analysis by Azizi et al. [10] and Hu Jiao et al. [9] (Fig. 3) showed that penile cancer with high NLR had a significantly worse lymph node invasion than patients with low NLR (OR 6.67, 95% CI 2.44, 18.22, p < 0.01). The heterogeneity between studies was non-significant, with an I^2^ value of 24% and 11% for univariate analysis and multivariate analysis, respectively. Therefore, the analysis model used for this outcome was fixed effects.

### 3.5 Meta-analysis results on the cancer-specific survival

**Fig. 4:**
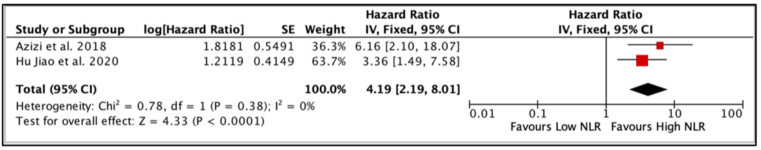
Forest plot comparison of unadjusted HR of CSS in penile cancer patients with high NLR versus low NLR.

**Fig. 5:**
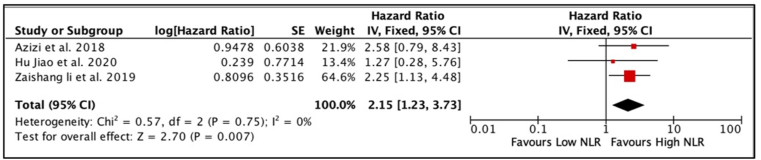
Forest plot comparison of adjusted HR of CSS in penile cancer patients with high NLR versus low NLR.

Three articles [9,10,16] with a total of 446 patients with penile carcinoma were analysed for CSS. According to the combined meta-analysis of univariate analysis, penile carcinoma patients with high NLR scores had significantly worse CSS than those with low NLR, with a HR of 4.19 (95% CI 2.19, 8.01, p = 0.01) (Fig. 4). Furthermore, the combined analysis of the three studies' multivariate analysis also revealed similar findings (HR 2.15, 95% CI 1.23, 3.73, p < 0.01) (Fig. 5). The chi-square and I^2^ tests used to analyse heterogeneity between studies revealed low heterogeneity (I^2^ = 0%). Thus, the fixed-effects model was selected.

### 3.6 Meta-analysis results on the overall survival

**Fig. 6:**
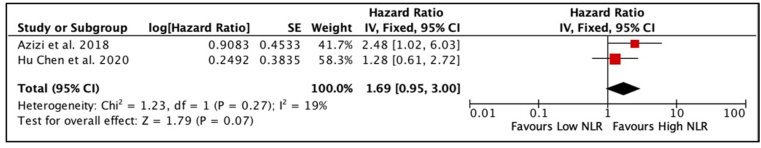
Forest plot comparison of adjusted HR of OS in penile cancer patients with high NLR versus low NLR.

Meta-analysis on the OS comprised two studies [10,12] with 309 penile cancer patients. Based on the forest plot results shown in Fig. 6, penile cancer patients with high NLR had a similar OS to those with low NLR (HR 1.69, 95% CI 0.95, 3, p = 0.07). The I^2^ index analysis on the forest plot shows no significant heterogeneity between the included studies (I^2^ = 19%), implying that the analysis model used for meta-analysis was fixed-model effects.

## 4 Discussion

The presence of lymph node involvement signifies locoregional cancer metastasis. Although the presence of metastatic disease in the lymph nodes has been found as an independent predictor of survival in penile cancer, lymphadenectomy for penile cancer does not always procure positive cancer cells and can be associated with false-negative results. Considering all these findings, the beneficial and harmful effects of lymph node examination should be cautiously interpreted. Studies have reported that PD-L1, squamous cell antigen, C-reactive protein, and P53 are all possible predictors for LNM in penile cancer [12]. Platelet to Lymphocyte Ratio (PLR) is another putative biomarker that can be detected by a simple complete blood count (CBC) examination [12]. NLR, on the other hand, has additional advantages as a useful predictor because it is affordable, repeatable, and can be utilized in remote locations.

NLR was found to be associated with LNM in this study, and it was also found to be an independent predictor for lymph node involvement. Forest plot analyses revealed that high NLR was associated with an increased probability of LNM. The Forest plot of the adjusted OR also showed that high NLR was an independent predictor for LNM. However, given that only two studies were included in this analysis, the conclusion should be taken cautiously.

Cancer patient with LNM has a poorer prognosis than those without the nodal disease. Tumor cells released substances that interact with stromal, myeloid, and lymphoid cells in primary tumors and the lymphatic system, lowering antitumor immunity and encouraging tumor growth, thus increasing the metastatic process [18]. The early detection of nodal invasion from the primary tumor is a critical point to adjust sufficient management. Previous studies have shown that NLR is a potential predictor for lymph node involvement during cancer progression. Higher pre-treatment NLR was identified as a predictor of LNM in endometrial cancers [19]. A study by Zhou et al. highlighted the role of NLR in association with LNM that is determinate on multivariable analysis (NLR ≥1.80) for patients with pancreatic neuroendocrine tumors and similarly in pancreatic ductal adenocarcinoma [20]. Xu et al. investigated that preoperative peripheral blood NLR concentration confers a higher risk of LNM in medullary thyroid carcinoma patients [21]. Other evidence of NLR as a useful complementary diagnostic tool for predicting pathological node involvement is observed in gastric cancer, notably because of its higher sensitivity and negative predictive value [22,23]. While most studies support the theory that NLR is associated with LNM, several studies demonstrated contradictive results. A study in 2016 by Maeda et al. discussed the lack of an association between NLR and PSA failure in prostate cancer patients who underwent Radical Prostatectomy. At the end of their study, they found no significant correlations of NLR with LNM (p = 0.062) [24]. A study by Yersal et al. (2017) about NLR and PLR in breast cancer subtypes also stated that there were no significant correlations between NLR or PLR and LNM count (p = 0.276). The different results of all this evidence should be cautiously interpreted, and the tumor burden might result in different tumor micro-environment and immune responses that affect NLR [25].

High NLR is also associated with worse outcomes in some malignancies, including urology malignancies. A meta-analysis by Wei et al. demonstrated that NLR was a predictor for worse CSS in bladder cancer, prostate cancer, and renal cell carcinoma [26]. Similar result by Shao et al. in renal cell carcinoma [27]. The Forest plot of this study showed that high NLR was an independent predictor for worse CSS in penile cancer. Nevertheless, our study shows no significant difference in OS based on NLR stratification. The latest study suggested that increasing pre-treatment neutrophil-to-lymphocyte ratio is related to the more severe clinical and pathological outcome as an independent predictor, including in testicular cancer. Staging of the tumors, the tendency to recurrence, and CSS are worse with higher NLR [28]. Higher post-orchiectomy NLR was found independently associated with recurrence in testicular cancer patients [29]. Among advanced/metastatic bladder cancer patients, NLR could independently improve the prediction of survival outcomes. Higher NLR is strongly linked to a poorer OS, with a HR of 5.06 in one study and the CSS of 36% lower in another study [30,31]. The patients with an NLR of ≤3.0 are likely to have better cancer outcomes and attain greater survival improvement from chemotherapy compared to the group with NLR of >3.0.

Several malignancies in different sites of system organs have been associated with poor clinical outcomes in the presence of increased NLR. A meta-analysis evaluating patients with breast cancer found a worse OS amongst greater NLR than the cut-off value [32]. An increased NLR indicated poor outcomes in patients with hepatocellular carcinoma, reflected in both OS and recurrence-free survival (RFS), according to one meta-analysis [33]. This finding has been confirmed in other prognostic studies on gastrointestinal cancer, head and neck cancer, and gynaecological cancer [[34], [35], [36], [37]]. Quantitative studies presented suggest an association between elevated NLR and poor clinical and survival outcomes across a wide spectrum of diagnoses, stages of the disease, and course of treatment.

However, several studies have found a different outcome pattern for NLR. Ojerholm et al. conducted a study about NLR as a bladder cancer biomarker, assessing prognostic and predictive values in SWOG 8710, and reported that NLR was neither a prognostic (p = 0.24) nor predictive (p = 0.86) biomarker for OS in muscle-invasive bladder cancer [38]. In contrast, Marchioni et al. demonstrated that a high NLR was associated with poorer oncological outcomes in patients affected by UTUC in terms of OS and RFS but not in cancer-specific survival (p = 0.77) based on a systematic review and meta-analysis study on high NLR as a prognostic factor in patients affected by Upper Tract Urothelial Cancer (UTUC) [39]. The different results of NLR as a prognostic factor in survival might be correlated to the indiscriminate role in tissue inflammation, the dichotomy of value measurement, and that most studies reporting NLR were observational in nature that were exposed to potential biases including the heterogeneous dataset.

The NLR has potential role to predict lymph node involvement in penile cancer. However, the difference in the NLR cut-off value was observed in this study. On the one hand, NLR indicators that are dichotomously distinct can have strong predictive significance for LNM and CSS, while in the other hand, the variations in cut-off values among published studies indicates that there are several variables which may influence the determination of NLR cut-off value. The determination of NLR mostly came from AUROC (Area Under Receiver Operating Characteristic) curve analysis. Each study assigned a different cut-off value based on ROC curve result which was influenced by study own's population. Since NLR value was derived from complete blood count examination, the patient's laboratory parameter and patient's demographic characteristic play significant role in affecting the result. The influence of race and age of the patients are important attributes in determination of NLR value. Furthermore, comorbidities and the degree of systemic inflammation in study's population may also contribute to the value.

Our systematic review and meta-analysis are not without limitations. First, all the included studies were retrospective in nature and will be susceptible to certain biases from a lack of standardized inclusion criteria, treatment schemes, and follow-up schedules. Second, there was no established (or standardised) cut-off value of NLR. Each study assigned a cut-off value with various methods based on the highest sensitivity and specificity from AUROC curve or used predefined cut-off values derived from other studies. Third, the population of this study are mostly Asian patients which does not represent the world population. Nonetheless, the inclusion of all published studies and critical analysis of the effect of NLR while controlling for other variables in LNM, CSS, and OS are strengths of this study. It is likely that large-scale prospective studies with a well-designed methodology are needed to establish the role of NLR in penile cancer.

## 5 Conclusion

This study demonstrated the value of NLR as an independent predictor for LNM and CSS in penile cancer. However, NLR in not proven to be an independent predictor for OS.

## Ethics committee approval

This systematic review and meta-analysis do not require an ethical approval.

## Funding source

None.

## Author contribution

• Haviv Muris Saputra (H.M.S.) is involved in the concept and project design, materials, literature search, data collection and/or processing, analysis and/or interpretation, writing the manuscript, and final approval of the version to be submitted.
• Furqan Hidayatullah (F.H.) is involved in the materials, literature search, data collection and/or processing, analysis and/or interpretation, writing the manuscript, and final approval of the version to be submitted.
• Yudhistira Pradnyan Kloping (Y.P.K.) is involved in the materials, literature search, data collection and/or processing, analysis and/or interpretation, writing the manuscript, and final approval of the version to be submitted.
• Johan Renaldo (J.R.) is involved in the concept and project design, supervision, resources, materials, literature search, data collection and/or processing, analysis and/or interpretation, writing the manuscript, and final approval of the version to be submitted.
• Eric Chung (E.C.) is involved in the supervision, resources, materials, writing the manuscript, and final approval of the version to be submitted.
• Lukman Hakim (L.H.) is involved in the concept and project design, supervision, resources, materials, literature search, data collection and/or processing, analysis and/or interpretation, writing the manuscript, and final approval of the version to be submitted.

## Guarantor

Lukman Hakim, Department of Urology, Faculty of Medicine, Universitas Airlangga, Universitas Airlangga Teaching Hospital, Indonesia. Email: lukman-h@fk.unair.ac.id.

## Informed consent

This systematic review and meta-analysis do not need an informed consent statement.

## Provenance and peer review

Not commissioned, externally peer-reviewed.

## Declaration of competing interest

The authors declare that they have no conflict of interest.
